# Nodular Melanoma in an African American Female

**Published:** 2019-04-09

**Authors:** Vincent Brown, Tom Reisler, Jake Laks

**Affiliations:** ^a^Department of Surgery, The Brody School of Medicine, East Carolina University, Greenville, NC; ^b^Division of Plastic and Reconstructive Surgery, Department of Surgery, The Brody School of Medicine, East Carolina University, Greenville, NC

**Keywords:** melanoma, nodular melanoma, skin lesion, skin cancer, exophytic mass

## Abstract

Melanoma incidence is increasing and is associated with the majority of skin cancer–related deaths. Most melanomas have an indolent growth and are difficult to diagnose, attributing to their increased risk of metastatic disease on initial presentation. Nodular melanoma accounts for roughly 15% to 30% of all the melanomas, making it the second most common melanoma. This case describes nodular melanoma, the atypical presentation, the diagnosis, and the treatment. This case is of special interest due to the rarity of the disease and this particular patient's disease presentation and management. Unfortunately, the patient refused further surgery, so definitive treatment was not achieved.

## CASE DESCRIPTION

**Figure 1:**
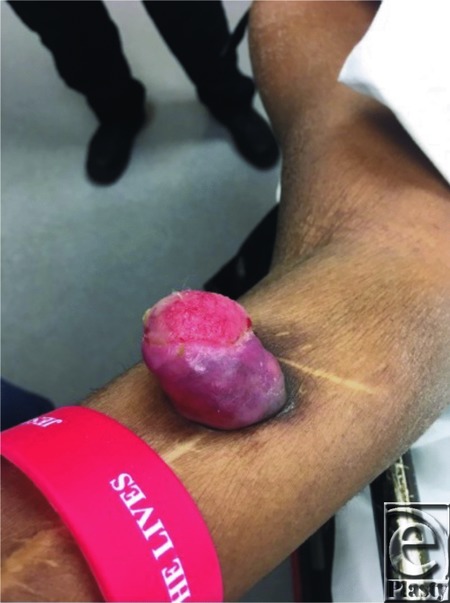
Exophytic mass of the right forearm.

A 63-year-old African American woman presented to the Plastic Surgery clinic from her nursing home with a 2-year history of a painful distal forearm mass. The lesion was initially thought to be a keloid from a stab wound more than 20 years ago; however, she decided to have it evaluated because of a recent increase in size and intermittent bleeding. She denied any numbness, tingling, or weakness of the hand. Her neurovascular examination had normal findings. Clinically, the mass was located on the volar aspect of the mid-forearm, was pink and ulcerated, and 3 × 3 × 4 cm in size (Fig 1). There was no associated supracondylar or axillary lymphadenopathy.

**Figure 2:**
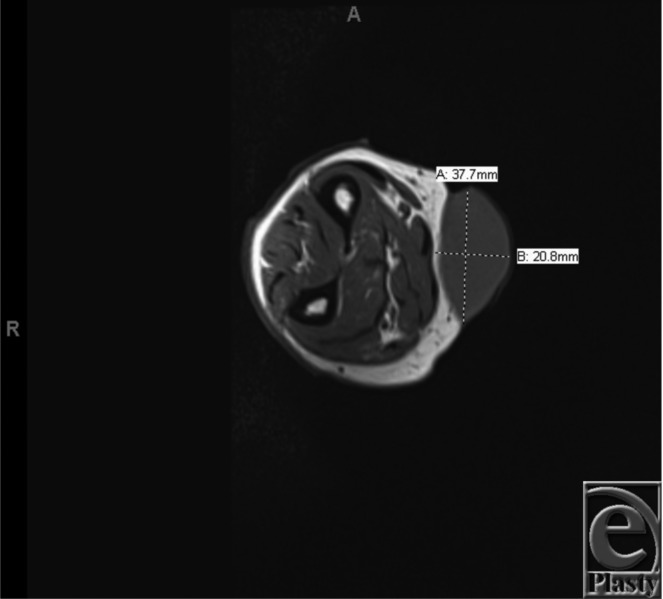
Magnetic resonance image of the right forearm showing the extent of the lesion.

Because of the unique characteristics of the mass, we further evaluated the lesion with a magnetic resonance image of the right forearm. This image demonstrated a 3.8 × 2.1 × 4.1-cm mass in the subcutaneous tissue without invasion into the fascia or muscle (Fig 2). The lesion was concerning for malignancy, so she was referred to a surgical oncologist for excisional biopsy. One month later, the mass was excised en bloc with careful dissection of the deep margin to maintain the fascia. We used a 6 × 4-cm elliptical incision, and this defect was closed primarily using suprafascial flaps for a tension-free closure.

On final pathology, the entire specimen was 6 × 4 × 0.8 cm. It was found to be an invasive nodular melanoma with positive, deep, and radial margins. This case was discussed at our multidisciplinary tumor board with plans of positron emission tomographic (PET) scan, reexcision, and sentinel lymph node biopsy.

Before her postoperative follow-up appointment, she underwent a PET scan, which unfortunately demonstrated a hyperactive node in the right axilla as well as a concerning left breast lesion. At her follow-up appointment, we offered a wide local excision with sentinel lymph node biopsy; however, the patient refused to have further surgery. On physical examination, there was a small, red, scaly lesion in the middle of the surgical site concerning for rapid recurrence. We referred her to a breast surgeon for workup of the left breast lesion, which ultimately was found to be ER/PR^+^ invasive lobular breast cancer. The patient was scheduled for close follow-up in both clinics but had not made her appointments. After reviewing the medical records, the patient refused to have any further surgical procedure at this time.

## QUESTIONS

1. What is the differential diagnosis of the mass?
2. What is nodular melanoma and how does it present clinically?
3. What is the workup of a skin lesion?
4. What is the management?

## DISCUSSION

For most skin lesions, physical presentation can aid in a correct diagnosis. The differential diagnosis in our patient included pyogenic granuloma, nodular basal cell carcinoma (BCC), squamous cell carcinoma (SCC), sarcoma, Merkel cell, and angioma. Each type of skin cancer has its own defining appearance, whether it be the pearly appearance of BCC or the scaly, red lesion seen with SCC. Typically, melanoma presents as an asymmetric, darkly pigmented lesion with irregular borders (ABCDE). However, the physical appearance of nodular melanoma differs, which helps distinguish it from other types of melanoma.^1^

**Figure 3:**
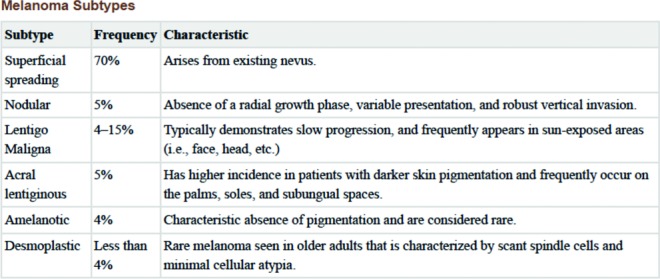
Melanoma subtypes and incidence. Used with permission from Ward et al.^2^

There are 4 major types of melanoma: superficial spreading, nodular melanoma, lentigo maligna, and acral lentiginous. Melanoma incidence is increasing and is associated with the majority of skin cancer-related deaths. Most melanomas have an indolent growth and are difficult to diagnose, attributing to their increased risk of metastatic disease on initial presentation. Nodular melanoma accounts for roughly 15% to 30% of all the melanomas, making it the second most common melanoma (Fig 3).^3^ These lesions are more prevalent in males older than 50 years and patients with fair skin and are commonly found on sun-exposed areas such as the head and neck. On examination, the nodular subtype can present as amelanotic, elevated, symmetrical, and firm on palpation. They are more aggressive and hold a poor prognosis because of their short radial growth phase and a more robust, vertical growth phase. A series of 96 patients demonstrated a correlation between nodular melanomas and poor prognostic features, including a deeper Breslow thickness, early ulceration, and higher mitotic rate.^4^

Similar to other skin lesions, diagnosis and staging of nodular melanoma are acquired using a thorough history and physical examination, tissue biopsy, sentinel lymph node dissections, and ancillary testing including imaging.^2^ For an actual tissue diagnosis, there are several ways to obtain a specimen, by punch, shave, or excisional biopsy. All techniques can offer valuable information, but shave biopsies can be prone to inadequate sampling. An excisional biopsy provides both a histological diagnosis and the possibility of complete excision and treatment of the skin lesion. Excisional biopsy is preferred in melanoma. Imaging, comprising preoperative computed tomography, magnetic resonance imaging, or PET, can be a key modality when further staging advanced disease. In this case, we evaluated the extent of the lesion using a magnetic resonance image of the right forearm (Fig 2). However, for the diagnosis of malignancy, a positive biopsy needs to be followed by complete excision of the lesion.

**Figure 4:**
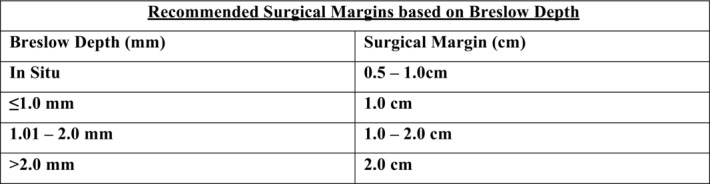
Recommended surgical margins based on the depth of the melanoma.

Once the diagnosis of nodular melanoma is confirmed, definitive treatment includes wide local excision with predetermined margins based on the Breslow depth (Fig 4). For clinically palpable nodes, or suspicious nodes identified on imaging (stage 3), a primary excision with sentinel lymph node biopsy is warranted. For more advanced stages, adjuvant therapy is administered on a case-by-case basis. These agents include interferon alfa and immune checkpoint inhibitors such as CTLA-4 and PD-1 antibodies.

## SUMMARY

Melanoma is increasing in incidence and should be within the differential diagnosis of a skin lesion. The workup includes a complete history and physical examination, a histological diagnosis, and ancillary testing to complete staging. For treatment, surgical excision is required with adjuvant therapies available on a case-by-case basis.
